# Asperosaponin VI suppresses ferroptosis in chondrocytes and ameliorates osteoarthritis by modulating the Nrf2/GPX4/HO-1 signaling pathway

**DOI:** 10.3389/fphar.2025.1539092

**Published:** 2025-02-28

**Authors:** Zhimeng Zhang, Daotong Yuan, Ximin Jin, Wenjie Chang, Yongkui Zhang, Wenpeng Xie

**Affiliations:** ^1^ First Clinical College, Shandong University of Traditional Chinese Medicine, Jinan, China; ^2^ Department of Orthopedic Surgery, Affliated Hospital of Shandong University of Traditional Chinese Medicine, Jinan, China

**Keywords:** Asperosaponin VI, osteoarthritis, ferroptosis, Nrf2/GPX4/HO-1 signaling pathway, chondrocyte

## Abstract

**Background:**

Asperosaponin VI (AVI) is a naturally occurring monosaccharide derived from *Dipsacus asperoides* renowned for its anti-inflammatory and bone-protective properties.

**Objective:**

To elucidate the specific mechanism through which AVI affects chondrocytes in osteoarthritis (OA).

**Methods:**

For the *in vitro* experiments, primary chondrocytes were to elucidate the molecular mechanisms underlying the action of AVI.For the *in vivo* experiments, rat OA models were established using a modified Hulth method. The severity of knee osteoarthritis was evaluated 8 weeks post-surgery. Micro-CT imaging, hematoxylin-eosin staining, and Safranin O-fast green staining were used to assess degeneration in rat knee joints. Immunohistochemistry techniques were conducted to measure the levels of collagen II, MMP13, Nrf2, GPX4, ACSL4, and HO-1 within cartilage tissues. ELISA assays were performed to measure those of TNF-α, IL -6, and PGE2 in serum samples.

**Results:**

AVI alleviated chondrocyte apoptosis and extracellular matrix degradation in rat OA induced by IL-1β. It attenuated the levels of TNF-α, IL-6, and PGE2 while reducing those of Fe^2+^ and malondialdehyde (MDA). AVI upregulated the expression of Nrf2, HO-1, and GPX4 while downregulating that of ACSL4. Mechanistic studies revealed that ML385-induced inhibition of the Nrf2 signaling pathway reversed the increase in GPX4 and ACSL4 expression and increased Fe^2+^ and MDA levels; treatment with erastin, a ferroptosis inducer, produced comparable results. *In vivo* experiments demonstrated that AVI improved the bone volume/tissue volume and trabecular separation values in OA rats; reversed the Osteoarthritis Research Society International score; upregulated Nrf2, HO-1, and GPX4 expression; downregulated ACSL4 and MMP13 expression, and decreased the serum levels of TNF-α, IL-6, and PGE2.

**Conclusion:**

Our findings suggest that AVI is a promising therapeutic agent for OA. It exerted its protective effect by regulating the Nrf2/GPX4/HO-1 signaling axis to inhibit cartilage cell ferroptosis and improve osteoarthritis.

## Introduction

Osteoarthritis (OA) is a prevalent chronic degenerative joint disease. In 2020, approximately 7.6% of the global population was affected by OA (Collaborators, 2023). According to the latest research projections, the prevalence of osteoarthritis (OA) is expected to rise from 48.6% to 95.1% globally by 2050. Among various forms of OA, knee osteoarthritis is the most prevalent, with a higher incidence in women compared to men (Siddiq et al., 2024).The understanding of OA has evolved from focal cartilage lesions to encompass damage across the entire joint, including the cartilage, subchondral bone, synovium, meniscus, ligaments, surrounding muscles, and fat pads (Li et al., 2022; Coaccioli et al., 2022; Tong et al., 2022; Yao et al., 2023) However, the current understanding posits that OA onset is characterized by cartilage erosion (Al-Hetty et al., 2023). Cartilage tissues comprise chondrocytes, which play a pivotal role in the pathogenesis of OA (Bernabei et al., 2023).

Ferroptosis exerts an influence on chondrocytes and contributes to OA pathogenesis. It is a form of regulated cell death that relies on iron and is driven by lipid peroxidation. In lipid peroxidation, the antioxidant system of glutathione deactivates peroxidized lipids, with glutathione peroxidase 4 (GPX4) as a crucial component of this system (Jiang et al., 2021). Consequently, GPX4 has been implicated in ferroptosis, and multiple studies have suggested that its regulation involves the Nrf2 signaling pathway (Wang et al., 2022; Xu et al., 2022; Ge et al., 2021). HO-1, an important downstream target of Nrf2 (Yang et al., 2022), plays a critical role in combating oxidative stress (Yang R. et al., 2024).

*D. asperoides*, a plant commonly used in traditional Chinese medicine for OA treatment (Tao et al., 2020), has been shown to exert anti-OA effects by reducing the levels of inflammatory markers, such as tumor necrosis factor-α (TNF-α), interleukin (IL)-1β (IL-1β), and IL-6 in serum (Hu et al., 2024), inhibiting the activities of various extracellular matrix (ECM)-degrading enzymes, including MMP13 (Chun et al., 2021a), and attenuating OA pathology progression (Chun et al., 2021b). AVI, an essential extract derived from *D. asperoides*, has been shown to exert anti-inflammatory and bone-protective propertie (Wei L. et al., 2023; Niu et al., 2023; Luo et al., 2024; Yang X. et al., 2024; Wei et al., 2024). These findings imply the therapeutic potential of AVI for OA.Therefore, this study aims to investigate the therapeutic effects of AVI on OA and explore its potential mechanisms of action. Our findings present a novel approach for treating OA and hold significant clinical and therapeutic implications.

## Materials and methods

### Cell culture and treatment

Primary chondrocytes were isolated from one-week-old Sprague–Dawley (SD) rats and cultured in Dulbecco’s Modified Eagle Medium supplemented with 5% fetal bovine serum and 1% penicillin/streptomycin. To establish a model of degenerative cartilage, the chondrocytes were stimulated with 10 ng/mL IL-1β (400-01B, Peprotech, America). The impact of AVI (PubChem CID: 14284436; HY-N0265, MedChemExpress, America) on the viability of primary chondrocytes was assessed using the Cell Counting Kit-8(CCK8) assay. To determine the optimal concentration, different concentrations of AVI were added to the degenerative cartilage. The Nrf2 signaling pathway inhibitor ML385 (HY-100253, MedChemExpress, America) was used to disrupt cellular processes. The ferroptosis inducer erastin (HY-15763, MedChemExpress, America) was used to trigger chondrocyte ferroptosis.

### Cell viability assay

Chondrocytes were cultured in the 96-well plates. Following the intervention, cells were incubated in a dark cell-culture incubator for 24 h.The CCK8 reagent (ES7011, Yishan, China) was added, bsorbance at 450 nm was measured using an enzyme-linked immunosorbent assay (ELISA) reader.

### Animal models

Animal experiments were approved by the Ethics Committee of the Affiliated Hospital of Shandong University of Traditional Chinese Medicine (Approval: SDSZYYAWE20240827001).

Eight-week-old male SD rats were randomly assigned to the following groups: sham operation (NC, n = 6), model (OA, n = 6), AVI low-concentration (AL, n = 6), AVI medium-concentration (AM, n = 6), AVI high-concentration (AH, n = 6), and positive control (PO, n = 6). OA was induced in rats using the modified Hulth method. After anesthetizing the rats with 3% pentobarbital sodium and confirming complete anesthesia, the medial collateral ligament was exposed on its inner side by disinfecting and opening the left knee joint. Subsequently, we incised it to free and remove the medial meniscus. The joint cavity was rinsed and sutured up layer by layer to prevent postoperative complications. The sham operation involved only the opening of the knee joint without any damage to ligaments or meniscus.

To establish osteoarthritis models, a 4-week period was allowed for unrestricted movement. Saline was administered via gastric lavage to both the sham operation and model groups; different concentrations of AVI were similarly administered to AVI-treatment groups. The PO group received celecoxib (12 mg/kg body weight) once daily via gastric lavage for 4 weeks.

Specimens from all experimental animals were collected following re-anesthetization with 3% pentobarbital sodium. Blood samples were obtained from the abdominal aorta using disposable collection needles, followed by centrifugation at 3,000 rpm for 10 min after standing at 26°C for 15 min. Then, blood serum samples were stored at −80°C for further analysis.

### Western blotting analysis

Cellular proteins were extracted using RIPA buffer (R0010, Solarbio, China) supplemented with PMSF. The protein concentration of each sample was determined using the BCA method (GK10009, Glpbio, America). Subsequently, the proteins were denatured with 5x protein loading buffer (GF 1811-10, Genefist, China), and a one-step PAGE quick preparation kit (GF 1820-10, Genefist, China) was utilized to prepare the polyacrylamide gel for electrophoresis. Following electrophoresis, transfer and blocking steps in sequence, primary antibodies including Collagen II (1:800; Affinity,AF0135 China), MMP13 (1:800; AF5355, Affinity, America), Nrf2 (1:500; GB115673, Servicebio, China), GPX4 (1:3,000; 67763-1-lg,Proteintech, America), ACSL4 (1:1000, GB115608, Servicebio, China), (1:600; GB15104, Servicebio, China), β-Actin (1:50000; 66009-1-lg, Proteintech, America) were diluted in universal antibody dilution solution (GF1600-02, Genefist, China) and incubated at 4°C overnight. After washing with TBST (Servicebio, G0004-500ML, China), corresponding secondary antibodies [HRP-conjugated goat anti-rabbit IgG (H + L) and HRP-conjugated goat anti-mouse IgG (H + L), GB23303, GB2330, Servicebio, China] were added and incubated at room temperature for 60 min. Finally, ECL substrate (MA0186-1, MeilunBio, China) was used to visualize the protein signals. ImageJ was used for grayscale quantification.

### qRT PCR

After the intervention, total RNA was extracted from cells using an RNA-Quick Purification Kit (RN001, Yishan, China). The purity and concentration of the extracted RNA were assessed before reverse transcription into cDNA using the Fast All-in-One RT Kit (RT001, Yishan, China). The reaction system was reconstituted following the instructions provided in the kit, with β-actin as an internal control. The expression of each target gene was quantified using the 2^−ΔΔCt^ method. The primer sequences are provided in the supplementary materials (Supplementary Figure S1A).

### Immunofluorescence analysis

Chondrocytes were isolated and seeded in 24-well plates at a density of 50,000 cells per well. After confirming cells adhesion, intervention was administered, followed by fixation using 4% paraformaldehyde (G1101-500ML, Servicebio, China) for 15 min and permeabilization using 0.3% Triton X-100 (IT9100, Solarbio, China). Subsequently, cells were incubated with a blocking solution containing 5% BSA at room temperature for 1 h, followed by incubation with primary antibodies against collagen II (1:200; AF0135, Affinity, America) and MMP13 (1:200; AF5355, Affinity, America) overnight at 4°C. This was followed by incubation with secondary antibodies in darkness at room temperature (1:400; RGAR004 and RGAR002, Proteintech, America) for 1 h. Cell nuclei were stained with DAPI at room temperature for 10 min and mounted onto glass slides with antifade mounting medium (P0-126-25mL, Beyotime, China). Finally, fluorescence intensity was measured.

### ELISA

ELISA kits were used to quantify TNF-α, IL-6, and PGE2 levels in cell-culture supernatants and rat sera following intervention. The following ELISA kits were used: Rat TNF-α High Sensitivity ELISA Kit (EK382HS-96, MultiSciences, China), Rat IL-6 ELISA Kit (JYM0646Ra, Wuhan Jiyinmei Biotech, China),and Rat Prostaglandin E2 (PGE2) ELISA Kit (JYM0446Ra, Wuhan Jiyinmei Biotech, China).The levels of Fe^2+^ and MDA in the cells post-intervention were determined using the Cell Ferrous Iron Colorimetric Assay Kit (E-BC-K881-M, Elabscience, China) and the MDA Colorimetric Assay Kit for Cell Samples (E-BCK0, Elabscience, China).

### Micro-CT analysis

The processed rat knee joints were scanned using a micro-CT instrument (Skyscan1276), and the resulting tomographic CT images were analyzed using CTAn and DataViewer.

### H&E and SO staining

The harvested knee joint specimens were initially fixed in 4% paraformaldehyde (Servicebio; G1101-500 ML) for a period of 2 days. The volume of the fixative was maintained at 15 times that of the knee joint, and the solution was replaced every 6 h. Subsequently, the specimens underwent decalcification using EDTA (Servicebio; G1105-500 ML) for 4 weeks. Following decalcification, the specimens were dehydrated, embedded in paraffin, and sectioned sequentially. Hematoxylin-eosin (H&E) staining was performed using the High-Definition Constant Staining Kit (G1076, Servicebio, China), while bone tissue was stained with Safranin O-Fast Green staining kit (G1053, Servicebio, China). All procedures were conducted strictly according to the manufacturer’s instructions. The degree of knee joint degeneration in each group was evaluated using the OARSI scoring system.

### Immunohistochemistry

Fixed with polyformaldehyde, the knee joint was decalcified using EDTA (G1105-500ML, Servicebio, China) and embedded in paraffin. It was sectioned and subjected to conventional immunohistochemistry using primary antibodies against collagen II (1:200; AF0135, Affinity, America), MMP13 (1:200; AF5355, Affinity, America), Nrf2 (1:500; GB115673, Servicebio, China), GPX4 (1:1000; 67763-1-lg, Proteintech, America), ACSL4 (1:1000; GB115608, Servicebio, China), and HO-1 (1:500; GB15104, Servicebio, China).

### Statistical analysis

*In vitro* experiments were independently conducted at least three times. Data were analyzed in GraphPad Prism 9.5 and are presented as the mean ± standard deviation ( $\bar{X}$ ± S). If the data met the assumptions of normal distribution and homogeneity of variances, one-way ANOVA was used for inter-group comparisons. Statistical significance was set at *P* < 0.05.

## Results

### AVI inhibited cartilage extracellular matrix degradation and suppressed inflammatory responses

**FIGURE 1:**
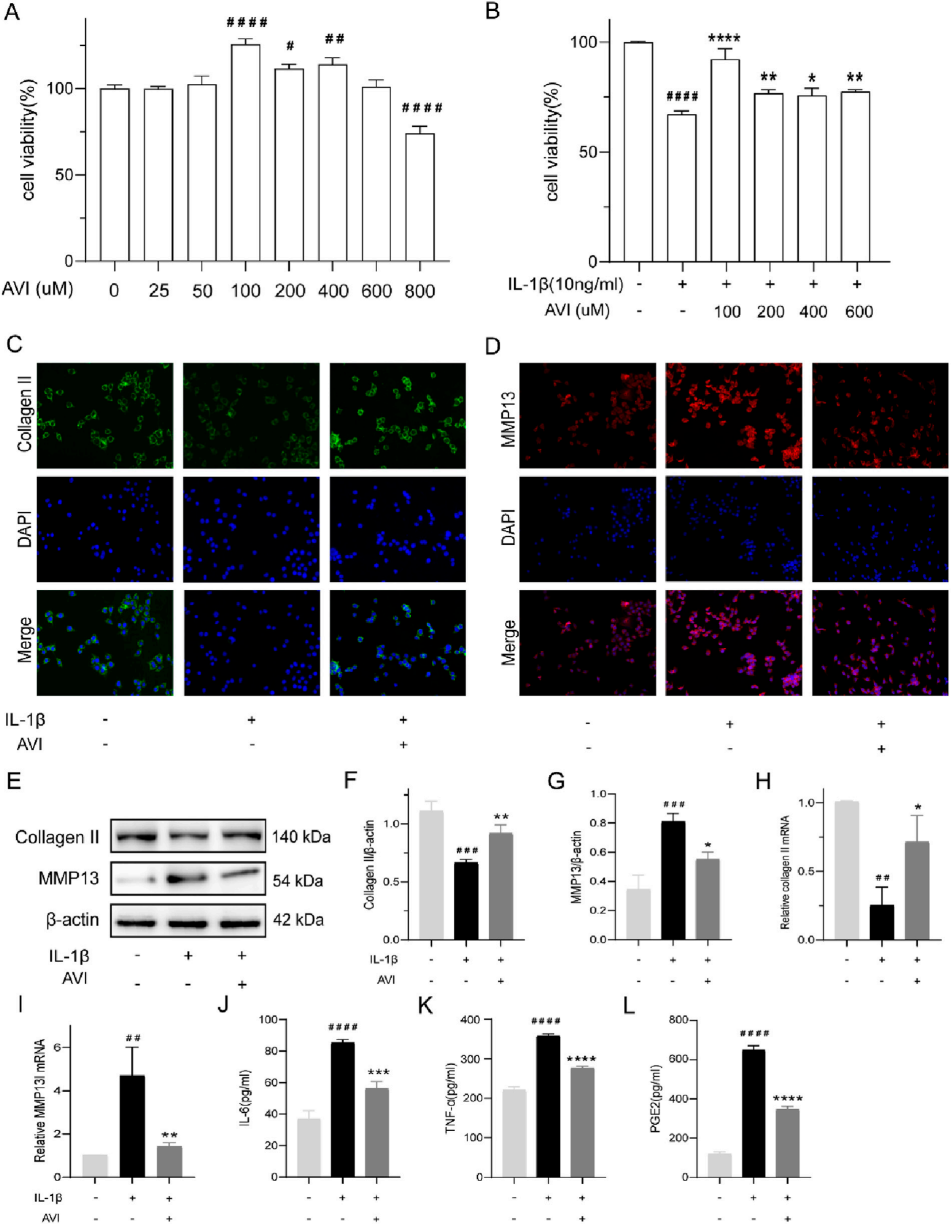
AVI inhibits the degradation of OA cartilage extracellular matrix and suppresses inflammatory responses. **(A)** The impact of various concentrations of AVI on rat chondrocyte viability. **(B)** The effect of different concentrations of AVI on IL-1ẞ-induced rat chondrocytes. **(C)** Immunofluorescence for Collagen II. **(D)** Immunofluorescence for MMP13. **(E–G)** Western blot and quantitative analysis for Collagen II and MMP13 expression levels. **(H, I)** qRT-PCR analysis for Collagen II and MMP13 gene expression. **(J–L)** ELISA results for IL-6, TNF-α, and PGE2 levels. All data are represented as mean ± SD (n = 3). Compared with the blank group.#p < 0.05,# #p < 0.01,# # #p < 0.001, # # # #p < 0.0001; compared with the OA model group, “p < 0.05, **p < 0.01, ***p < 0.001, ****p < 0.0001.

To investigate the *in vitro* mechanism of action of AVI, We validated the morphology of chondrocytes using light microscopy and toluidine blue staining techniques. (Supplementary Figures S1B, C). Within the concentration range of 0–600 uM, AVI did not exhibit significant cytotoxicity (Figure 1A). Against IL-1β-induced OA chondrocytes, at a concentration of 100 uM, AVI effectively reversed the decrease in cell viability caused by IL-1β (Figure 1B). Therefore, 100 uM AVI was selected for further experiments.

Collagen II expression was reduced, whereas that of MMP13 was enhanced in IL-1β-induced OA cells. Immunofluorescence analysis showed that AVI decreased MMP13 accumulation in OA chondrocytes and increased Collagen II expression (Figures 1C, D). Similar results were obtained in Western blotting and PCR analyses (Figures 1E–I). AVI reduced TNF-α, IL-6, and PGE2 levels in OA chondrocytes (Figures 1J–L).

### AVI suppressed ferroptosis and conferred protection to OA cartilage

**FIGURE 2:**
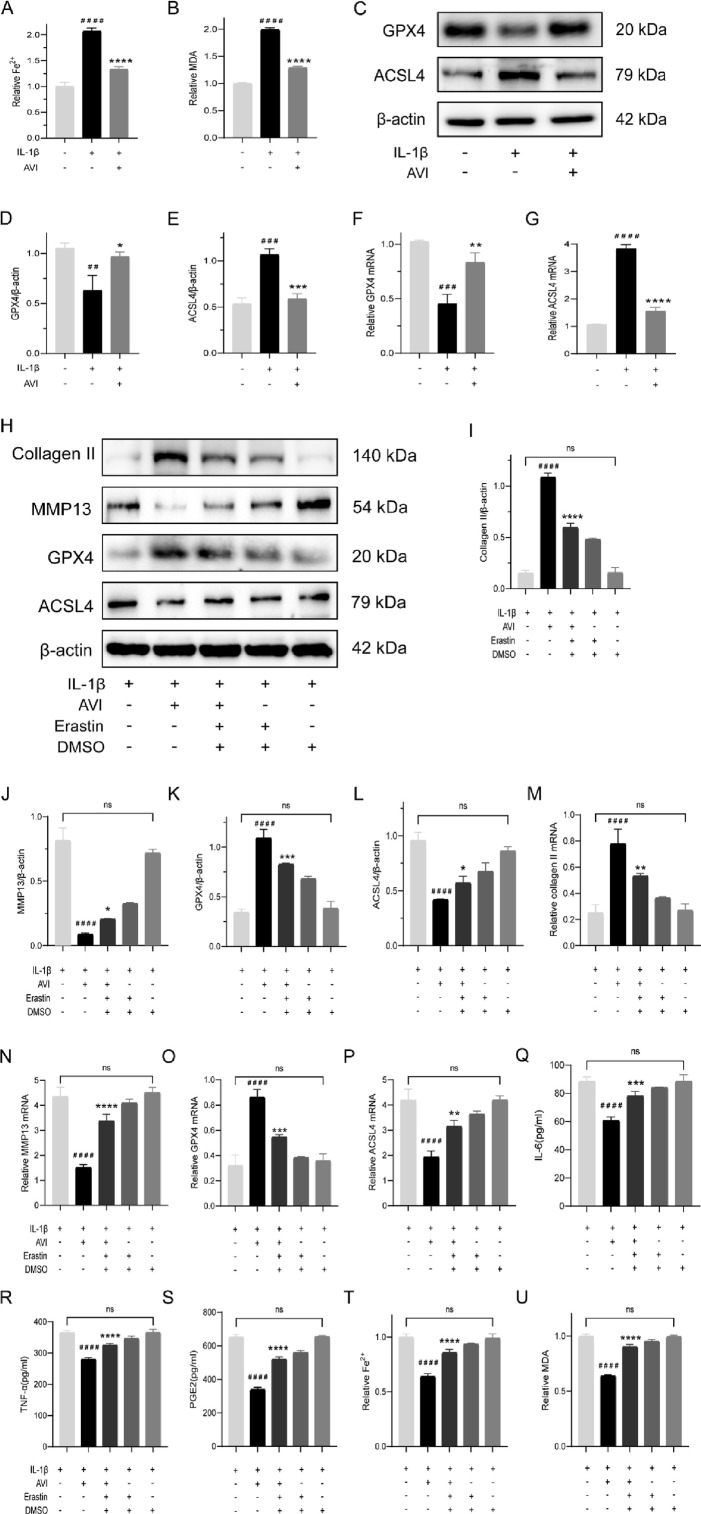
AVI suppresses ferroptosis and confers protection to OA cartilage. **(A)** Fe^2+^ content in each group. **(B)** MDA content in each group. **(C–E)** Western blot and quantitative analysis of GPX4 and ACSL4 expression levels. **(F–G)** qRT-PCR analysis of GPX4 and ACSL4 gene expression. **(H–L)** Western blot and quantitative analysis for Collagen II,MMP13,GPX4 and ACSL4 expression levels. **(M–P)** qRT-PCR analysis for Collagen II,MMP13,GPX4 and ACSL4 gene expression. **(Q–S)** ELISA results for IL-6, TNF-α, and PGE2 levels. **(T)** Fe^2+^ content in each group. **(U)** MDA content in each group. All data are represented as mean ± SD. (n = 3). Compared with the blank group,#p < 0.05,##p < 0.01,# # #p < 0.001,# # # #p < 0.0001; compared with the OA model group. *p < 0.05, **p < 0.01, ***p < 0.001, ****p < 0.0001.

AVI effectively reversed the elevated expression of Fe^2+^ and MDA in OA chondrocytes (Figures 2A, B). Western blotting analysis showed that AVI reversed the decreased expression of GPX4 and increased that of ACSL4 in OA chondrocytes (Figures 2C–E). Similar results were obtained by qRT-PCR (Figures 2F,G).

We investigated the potential of AVI to inhibit ferroptosis and protect OA chondrocytes by treating AVI-treated OA chondrocytes with erastin (a ferroptosis inducer). The effects of AVI on upregulating collagen expression and downregulating MMP13 expression were reversed following erastin treatment. Alterations in the expression of GPX4 and ACSL4, were also observed (Figures 2H–L). These findings were validated by qRT-PCR results (Figures 2M–P). Moreover, we observed a notable elevation in the levels of TNF-α, IL-6, and PGE2 (Figures 2Q–S). Erastin intervention elevated Fe^2+^ and MDA levels in OA chondrocytes (Figures 2T, U).

### AVI attenuated ferroptosis in chondrocytes by modulating the Nrf2/HO-1/GPX4 signaling axis

**FIGURE 3:**
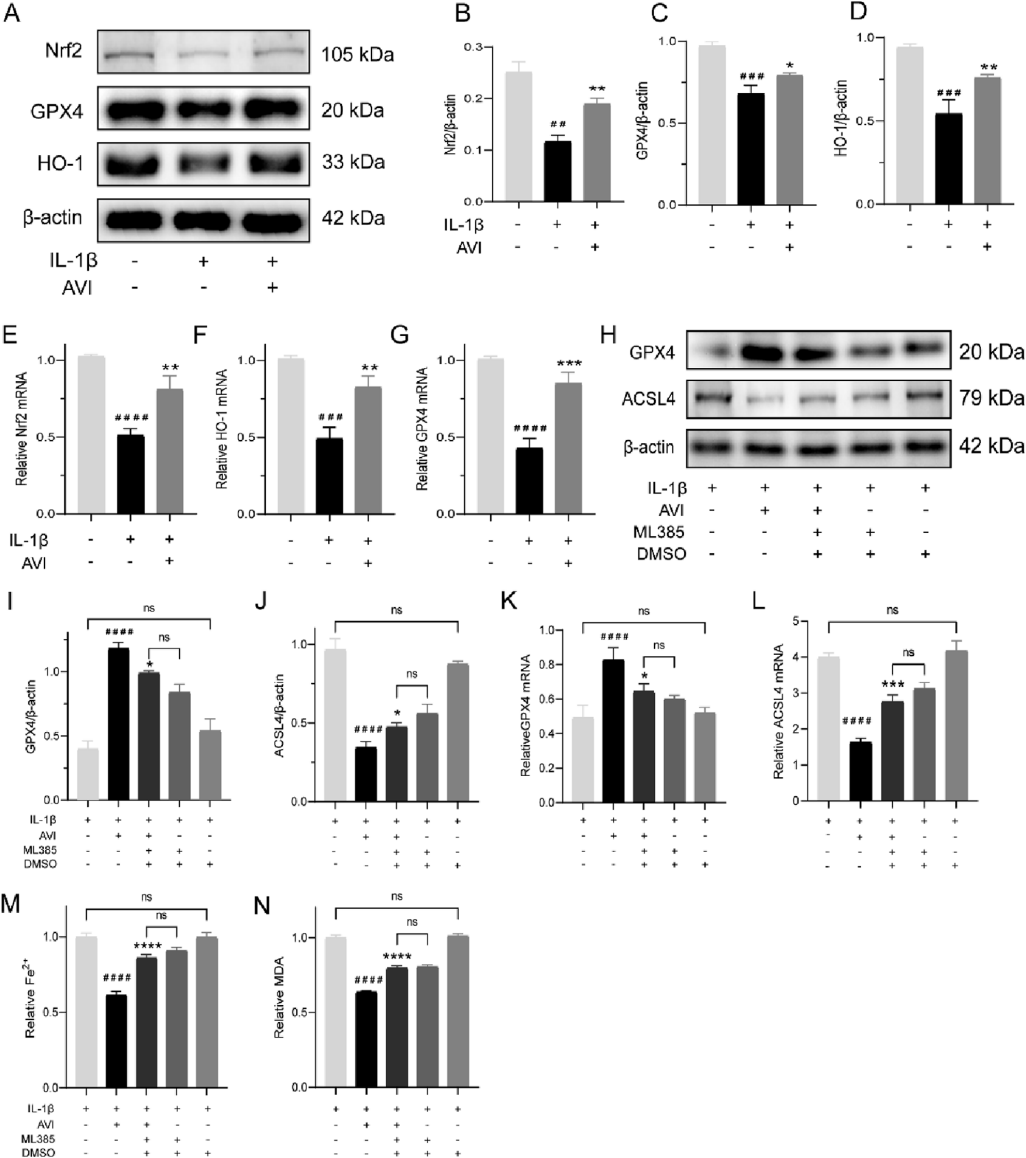
AVI attenuated ferroptosis in chondrocytes by modulating the Nrf2/HO-1/GPX4 signaling axis. **(A–D)** Western blot and quantitative analysis for Nrf2,GPX4 and HO-1 expression levels. **(E–G)** qRT-PCR analysis for Nrf2,GPX4 and HO-1 gene expression. **(H–J)** Western blot and quantitative analysis for GPX4 and AVSL4 expression levels. **(K, L)** qRT-PCR analysis for GPX4 and ACSL4 gene expression. **(M)** Fe^2+^ content in each group. **(N)** MDA content in each group.All data are represented as mean ± SD. (n = 3). Compared with the blank group. #p < 0.05,##p < 0.01,# # #p < 0.001,# # # #p < 0.0001; compared with the OA model group, *p < 0.05, **p < 0.01, *p < 0.001, ***p < 0.0001.

We further elucidated the regulatory mechanism of AVI on ferroptosis. Western blotting analysis showed the decreased levels of Nrf2, GPX4, and HO-1 proteins in OA chondrocytes; however, they increased following AVI treatment (Figures 3A–D).These findings were confirmed by qRT-PCR (Figures 3E–G).

We observed a reversal in the increased GPX4 expression and decreased ACSL4 expression induced by AVI upon ML385 treatment (Figures 3H–J). Consistent results were obtained by qRT-PCR analysis (Figures 3K, L). Following ML385 intervention, we observed an increase in the levels of Fe^2+^ and MDA in OA chondrocytes (Figures 3M, N).

### AVI mitigated OA progression in rats via the Nrf2/GPX4/HO-1 signaling axis

**FIGURE 4:**
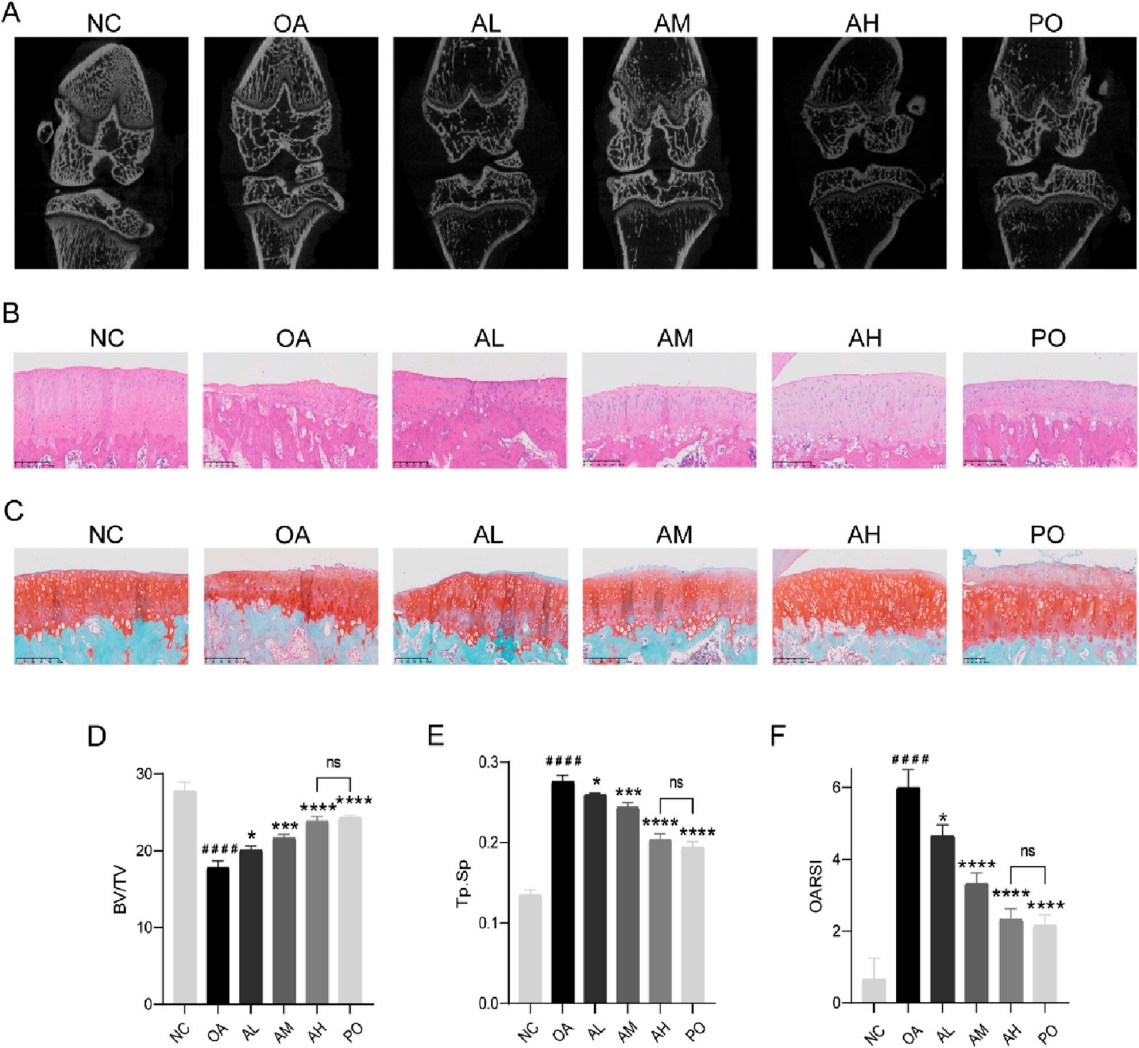
AVI mitigated OA progression in rats via the Nrf2/GPX4/HO-1 signaling axis. **(A)** micro-CT images of knee joints. **(B)** H&E staining. **(C)** Safranin O-fast green staining. **(D, E)** BV/TV and Tb.Sp values. **(F)** OARSI scores. All data are represented as mean ± SD. (n = 3). Compared with the blank group.#p < 0.05,# #p < 0.01,# # #p < 0.001,# # # #p < 0.0001; compared with the OA model group, *p < 0.05, **p < 0.01, ***p < 0.001, ***p < 0.0001.

Compared with those in the NC group, rats in the OA group had significantly narrowed joint space and visible osteophytes around the joint area, abnormal trabecular bone structure and reduction, decreased BV/TV value, and increased Tb.Sp value. However, following AVI treatment, there was a dose-dependent improvement in OA manifestations without significant differences in BV/TV or Tb.Sp values between the AH and PO groups (Figures 4A, D, E). In contrast to the NC group, the OA group showed roughness of the joint surface, presence of severe area cracks, disordered cell arrangement, and indistinguishable structural layers. SO staining revealed shallow and uneven staining in the OA group, with differences compared to the surrounding tissue cartilage staining in the NC group. OARSI scores were significantly higher in the OA group than in the NC group. However, following AVI intervention, we observed a dose-dependent reversal in the increase in OARSI scores and no significant difference between AM and PO groups. Furthermore, HE and SO staining results showed smoother joint surfaces, with normal cell arrangement and distinguishable structural layers, and normalized cartilage surrounding stained tissue sections (Figures 4B, C, F).

**FIGURE 5:**
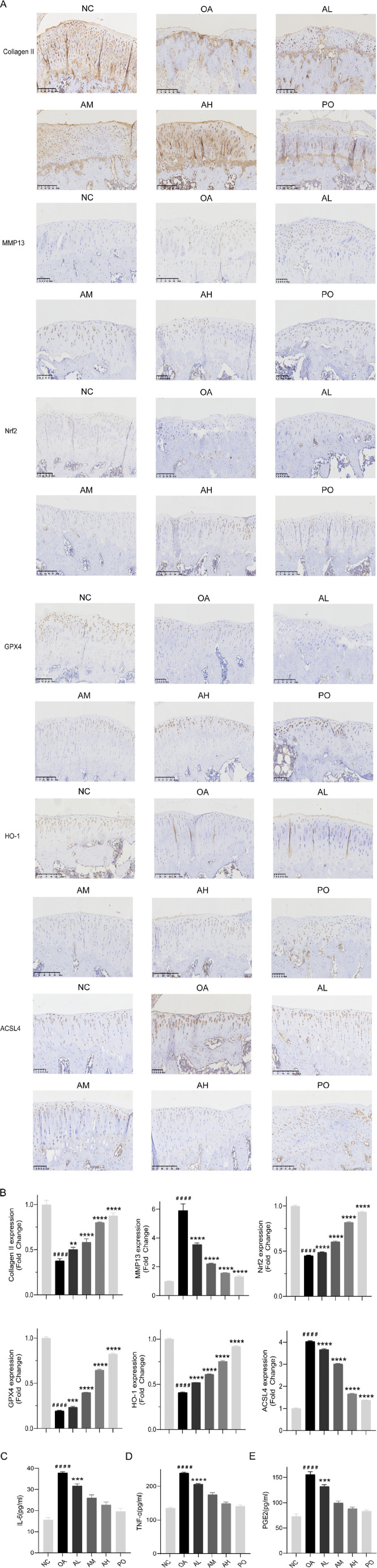
AVI mitigated OA progression in rats via the Nrf2/GPX4/HO-1 signaling axis. **(A)** IHC of Collagen II, MMP13, Nrf2, GPX4, HO-1, and ACSL4. **(B)** Relative quantitative analysis of expression of Collagen II, MMP13, Nrf2, GPX4, HO-1, and ACSL4. **(C–E)** ELISA results for IL-6, TNF-α, and PGE2 levels.

**FIGURE 6:**
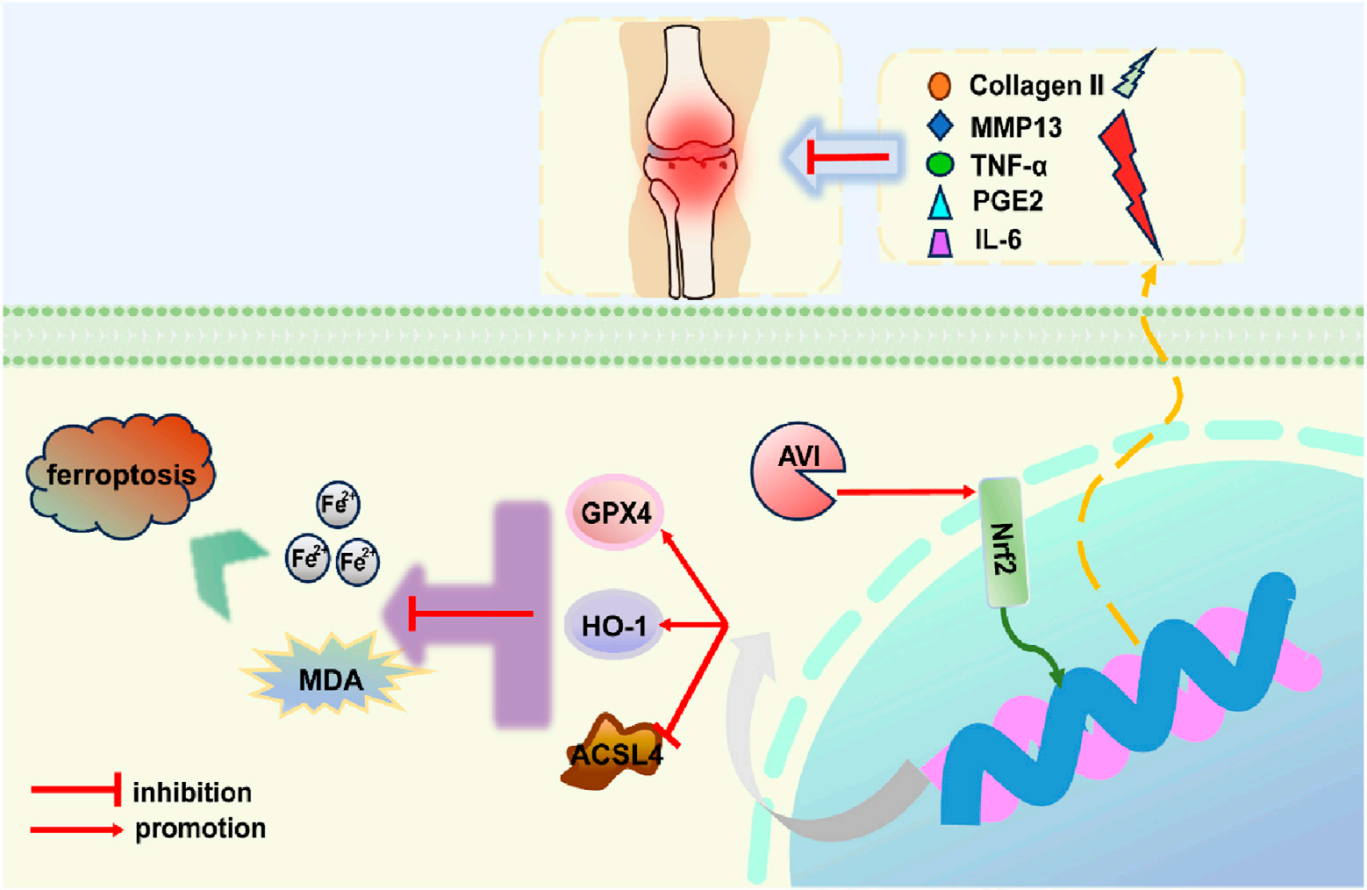
Concise mechanism diagram of AVI.

IHC results revealed a decrease in the expression of collagen II, Nrf2, GPX4, and HO-1 in the model group and an increase in that of MMP13 and ACSL4. However, following AVI intervention, these effects were reversed (Figures 5A, B). The levels of TNF-α, IL-6, and PGE2 in rat serum were assessed, and AVI mitigated the increase in the levels of inflammatory factors in the OA group (Figures 5C–E). These findings suggest that AVI may modulate the Nrf2/GPX4/HO-1 signaling axis to attenuate OA progression. In conclusion, the aforementioned evidence demonstrates that AVI modulates the Nrf2/GPX4/HO-1 signaling axis to suppress chondrocyte ferroptosis and ameliorate OA (Figure 6).

## Discussion

OA, a prevalent degenerative joint disease among the elderly, imposes a substantial economic burden and is associated with various serious consequences, including disability, cardiovascular complications, and heightened risk of depression and suicide (Hunter et al., 2020; Wang et al., 2021; Leifer et al., 2022; Zhao et al., 2023). Consequently, the detrimental impact of OA on both individuals and society is expected to increase in the future (Scheuing et al., 2023). Therefore, it is imperative to implement effective interventions for OA to mitigate its progression (Whittaker et al., 2021; Hawker and King, 2022).

As the sole cell type in cartilage tissue, chondrocytes maintain homeostasis by producing the extracellular matrix (Adam et al., 2024; Zhao et al., 2024). Traditional understanding posits that cartilage degeneration leads to OA; however, recent insights indicate that inflammatory mediators produced by the synovium, cartilage, and subchondral bone ultimately cause cartilage damage (Motta et al., 2023).Increasing evidence suggests that inflammation plays a major role in OA (De Roover et al., 2023; Knights et al., 2023; Wei G. et al., 2023; Bradley et al., 2023).Chondrocytes associated with OA actively secrete inflammatory cytokines (Molnar et al., 2021), including IL-1β and TNF-α, and their sustained release stimulates the production of MMP13 (Rim et al., 2020), which is implicated as pivotal in type II collagen degradation in OA (Chisari et al., 2020; Yunus et al., 2020). Additionally, their interaction not only enhances each other’s production, but also elevates the expression of other pro-inflammatory cytokines and intermediates, such as IL-6 and PGE2 (Kapoor et al., 2011; Sampath et al., 2023).

AVI, a single herbal compound extracted from *D. asperoides*, demonstrates anti-osteoarthritis effects akin to those of *D. asperoides*. Multiple studies have indicated that AVI effectively inhibits inflammatory responses (Jiang et al., 2022; Pang et al., 2022; Zhang et al., 2020). Our experiments have confirmed that AVI significantly reduced the expression of IL-6, PGE2, and TNF-α in OA cartilage, suggesting its potential for alleviating the inflammatory response in OA. It is widely acknowledged that immune cells are involved throughout the entire inflammatory process. Immune cells, represented by macrophages in the synovium, profoundly affect the inflammatory response. Under diverse conditions, they differentiate into M1-type macrophages that promote inflammation and M2-type macrophages that inhibit inflammation (Zheng et al., 2024).Previous studies have demonstrated that AVI significantly inhibits M1 polarization and promotes polarization towards the anti-inflammatory M2 phenotype in macrophages (Luo et al., 2022). These findings are consistent with our results, suggesting that AVI may modulate inflammation by influencing macrophage polarization.

Ferroptosis has been implicated in OA progression (Zhu et al., 2023), and its mechanism is believed to stem from the failure of the glutathione-dependent antioxidant defense system (Xiong et al., 2022). More and more evidence suggests that GPX4 and ACSL4 play an extremely important role in ferroptosis (Chen et al., 2021; Yao et al., 2021). Additionally, MDA and Fe^2+^ act as markers of ferroptosis (Cruz-Gregorio and Aranda-Rivera, 2023). The latest evidence suggests that Nrf2 could modulate GPX4 and HO-1 expression (Guo et al., 2022). Our findings validated that AVI can activate Nrf2 to upregulate GPX4 and HO-1 expression, downregulate ACSL4 expression, and reduce MDA and Fe^2+^ levels, while saving the expression of type Collagen II. The accumulation of inflammatory cytokines, such as IL-1β and TNF-α, may lead to increased iron uptake and accumulation, thereby inducing ferroptosis under inflammatory conditions (Feng et al., 2020). AVI’s anti-inflammatory effect indirectly confirms its potential for alleviating OA-induced ferroptosis. This finding is consistent with other research (Wei et al., 2024).*In vitro* experiments, DMSO is inevitably used. Although the experimental design has been relatively perfect, it may inevitably affect the experimental results.

*In vivo*, to assess the protective effect of AVI on OA, we established a rat OA model. AVI ameliorated joint space narrowing in OA. Additionally, histological analyses, including H&E and SO staining, revealed that AVI improved cellular arrangement and attenuated cartilage degradation in OA. Furthermore, HIC and ELISA assays indicated that AVI activated the Nrf2/GPX4/HO-1 signaling pathway to suppress ferroptosis and inflammatory responses in OA cartilage. Therefore, our research results indicate that the AVI can slow the progress of OA.

## Conclusion

*In vivo* and *in vitro* test results suggest that AVI modulates the Nrf2/GPX4/HO-1 signaling pathway to inhibit cartilage cell ferroptosis and alleviate osteoarthritis. Our findings showed AVI as a promising therapeutic agent.

## Limitations

This study has certain limitations. The direct verification of the interaction between the Nrf2/GPX4/HO-1 signaling pathway was not conducted, and there are also inadequacies in verifying the ferroptosis mechanism. We plan to address these shortcomings in future experiments.

## Data Availability

The original contributions presented in the study are included in the article/Supplementary Material, further inquiries can be directed to the corresponding authors.
