# Supplementary material for: Asperosaponin VI suppresses ferroptosis in chondrocytes and ameliorates osteoarthritis by modulating the Nrf2/GPX4/HO-1 signaling pathway
Source: Front Pharmacol. 2025 Feb 28;16:1539092. doi: 10.3389/fphar.2025.1539092 (PMC11906723; doi:10.3389/fphar.2025.1539092)
Supplement: Supplementary file 1 [file Image1.pdf]

A

Primer sequence

Gene	Forward primer (5'–3')	Reverse primer (5'–3')
Nrf2	AACACAAGAGCCCCTGTGTGGC	TGCCCCTGAGATGGTGACAA
GPX4	GAACTTCACCAAGTTCCTCATCG	TGGGGCAGGTCCTTCTCTATC
HO-1	CAGCATGTCCCAGGATTTGTC	CCTGACCCTTCTGAAAGTTCCTC
ACSL4	TTCTTCTCCGCTTACACTCTCTG	ATTCATCTCTTGGACTTTGCTCAT
Collaagen II	ACACCGCTAACGTCCAGATG	GTACGTGAACCTGCTGTTGC
MMP13	TGATGCTAACCAGACTATGGACAA	ATGACTCTCACAATGCGATTACTC
ACTIN	CCACGAAACTACCTTCAACTCC	TCTTGATCTTCATTGTGCTGGG

B

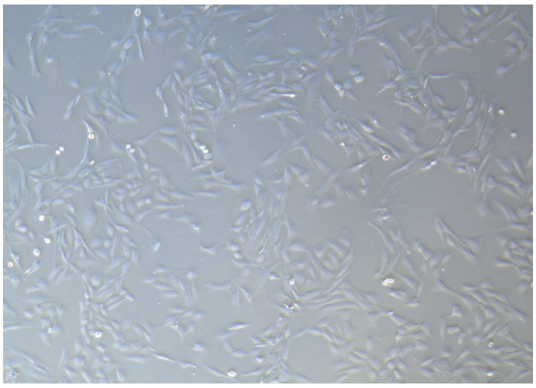

C

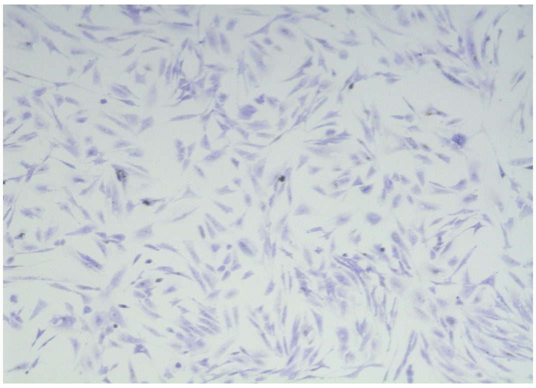

FigS1. A.Primer sequence.B.Primary rat articular chondrocytes were observed under an optical microscope. C.Toluidine blue stains of Primary Rat Chondrocytes for Enhanced Visualization.
